# Valorization of Plant Biomass Through the Synthesis of Lignin-Based Hydrogels for Drug Delivery

**DOI:** 10.3390/gels12020104

**Published:** 2026-01-27

**Authors:** Natalia Cárdenas-Vargas, Nazish Jabeen, Jose Huerta-Recasens, Francisco Pérez-Pla, Clara M. Gómez, Maurice N. Collins, Leire Ruiz-Rubio, Rafael Muñoz-Espí, Mario Culebras

**Affiliations:** 1 Institute of Materials Science (ICMUV), University of Valencia, c/Catedrátic José Beltrán 2, 46980 Paterna, Spain; natalia.cardenas@uv.es (N.C.-V.); nazish.jabeen@uv.es (N.J.); jose.huerta@uv.es (J.H.-R.); rafael.munoz@uv.es (R.M.-E.); 2 Stokes Laboratories, School of Engineering, Bernal Institute, University of Limerick, V94 T9PX Limerick, Ireland; 3 Macromolecular Chemistry Group (LQM), Physical Chemistry Department, Faculty of Science and Technology, University of the Basque Country (UPV/EHU), 48940 Leioa, Spain

**Keywords:** lignin, hydrogels, drug release

## Abstract

This study focuses on obtaining lignin-based hydrogels from pruning residues of orange trees in the Safor region (Valencia) using an alkaline extraction method. The structural analysis of the obtained lignin was carried out using Fourier-transform infrared spectroscopy (FTIR), which revealed the characteristic functional groups of lignin, as well as its structural monolignols: syringyl and guaiacyl. The thermal properties were analyzed using differential scanning calorimetry (DSC) and thermogravimetric analysis. The DSC thermogram revealed a relatively low glass transition temperature (*T*_g_) of 67 °C, which may be attributed to partial lignin chain degradation during alkaline extraction. Soda lignin was obtained at 190 °C with an approximate yield of 10.8% relative to the initial biomass and subsequently used to synthesize poly(vinyl alcohol) (PVA)-based hydrogels for ibuprofen encapsulation. Finally, the release experiments of the encapsulated ibuprofen were carried out in an aqueous phosphate buffer medium (pH = 7) at room temperature. A multi-curve response analysis (MCR) algorithm using the Korsmeyer–Peppas (KP) concentration model was used to analyze the release curves, which concluded that the drug and water-soluble lignin fraction (SLF) were released at different rates. For both components, a good correlation was obtained between the measured responses and those provided by the KP model. The release profile indicated that approximately 87% of the initial ibuprofen load was released from the hydrogel within 5 h, highlighting the promising potential of lignin-based hydrogels for drug delivery applications.

## 1. Introduction

The circular economy has become a fundamental paradigm in the development of sustainable solutions to today’s pressing environmental challenges. Beyond waste reduction and reuse, it promotes the transformation of traditionally discarded materials into value-added resources, fostering more efficient, resilient economic systems. In fact, it is estimated that the proper implementation of waste management strategies could lead to a reduction of up to 70% in global GHG emissions [1]. Among the most promising waste streams for circular valorization are agricultural residues, which reach nearly 998 million tons per year. In Spain alone, this figure is around 27 million tons, with approximately 80% corresponding to organic matter rich in lignocellulosic biomass [2,3,4,5], which is emerging as an invaluable resource to produce bioproducts and bioenergy [6,7]. The structural architecture of lignocellulosic biomass is defined by a complex assembly of cellulose, hemicellulose, and lignin [8]. These biopolymers are intricately linked through an extensive network of hydrogen bonding and cross-linking, resulting in a highly recalcitrant and resilient matrix. The mechanical integrity of plant cell walls is largely attributed to cellulose, a linear chain of β-1,4-linked glucose units. Beyond its biological role, this biopolymer is a critical industrial feedstock utilized in the manufacture of paper and biofuels, as well as the synthesis of organic acids, nanocellulose, and various pharmaceutical products [9]. Hemicellulose, by contrast, is an amorphous, branched polymer composed of pentoses and hexoses. It acts as a matrix that binds cellulose fibers, offering flexibility and enhancing wall interactions. Though less studied than cellulose, hemicellulose has significant potential for producing furfural and biogas [10]. Lignin, accounting for 10–15% of lignocellulosic biomass, is a complex, highly cross-linked polymer composed of phenylpropanoid units [11]. It acts as an adhesive between cellulose and hemicellulose and contains a broad range of functional groups [12]. The global lignin reservoir is estimated at over 300 billion tons, increasing by 20 billion tons annually. The global pulp and paper sector generates an estimated 50 million tons of lignin annually, primarily as a residual byproduct of the pulping process [13]. Additionally, lignin production is expected to increase due to the growing interest in lignocellulosic biorefineries. Despite its abundance and high functionality, most lignin is currently burned for energy recovery, and only a small fraction is isolated for material or chemical applications [14,15,16]. Therefore, the efficient and sustainable use of this polymer is more relevant than ever.

Due to the presence of phenolic groups in its molecule, lignin has been extensively explored in the production of carbon fibers, adhesives, and resin [17,18,19]. More recently, its antioxidant, antimicrobial, UV-protective, and biocompatible properties have positioned lignin as a promising component for advanced and biomedical materials [20]. It has been employed in the design of drug delivery systems, such as nanoparticles, micelles, emulsions, and especially hydrogels [21,22]. Lignin-based hydrogels are of particular interest due to their porosity, water-swelling capacity, and responsiveness to pH and temperature, making them suitable for tissue engineering, wound healing, and controlled drug release [23,24,25]. Their porous structure mimics the extracellular matrix, while their tunable properties allow precise control over drug diffusion, positioning them as smart, nature-inspired biomaterials for pharmaceutical applications [26,27,28].

This study provides novel insights into lignin-based hydrogels produced from orange tree pruning residues from the Safor region (Valencia), which serve as a renewable lignocellulosic source of lignin. The extraction used was the alkaline method, selected for its higher yield and process simplicity. Moreover, the extracted lignin was characterized by Fourier-transform infrared spectroscopy (FTIR), thermogravimetric analysis (TGA), and differential scanning calorimetry (DSC) to assess its structure and thermal behavior. The characterized lignin was then incorporated into poly(vinyl alcohol) (PVA)-based hydrogels specifically designed for the loading and controlled release of ibuprofen. To evaluate drug release, we employed a combined Multivariate Curve Resolution and Korsmeyer–Peppas framework. This approach allows us to deconvolve overlapping release profiles, enabling a distinct kinetic evaluation of both the ibuprofen and the water-soluble lignin fraction, in contrast to what is typically reported. This methodology offers deeper mechanistic insights than single-component models. By independently modeling the release of both the drug and the soluble lignin, we demonstrate a pathway for the rational design of multifunctional platforms capable of precise co-release or sequential delivery. These systems hold significant promises for wound dressings and topical anti-inflammatory therapies. In such applications, the sustained delivery of ibuprofen can work in tandem with the innate antioxidant and bioactive properties of lignin to produce synergistic therapeutic effects. Ultimately, by utilizing sustainably sourced and readily extractable biopolymer, this work positions these hydrogels as a resource-efficient, high-value alternative to conventional synthetic polymer systems in the pharmaceutical and biomedical sectors.

## 2. Results and Discussion

The soda lignin was successfully extracted from orange tree pruning residues, yielding approximately 10.8% of the initial biomass, which is consistent with the approximate lignin content of citrus pruning residues (~20%) [29]. Reported yields for soda lignin extraction vary widely depending on biomass type and process conditions. For example, soda pulping of non-woody residues such as Miscanthus sinensis has produced lignin yields ranging from ~10% to 40% depending on pH and acidification conditions, illustrating that yield is highly responsive to process parameters and biomass characteristics [30]. Similarly, soda lignin extraction from oil palm empty fruit bunch fiber has been reported to yield ~15% under standard pulping conditions [31]. Technical reviews further indicate that soda lignin processes can achieve high overall yields (>80%) under optimized or industrial conditions [32], although such values generally require process configurations, solvent systems, or post-treatments that differ substantially from simple laboratory soda extraction. In our case, the temperature employed (190 °C) constitutes a key difference compared to other soda processes, and contributes to reduced extraction yield, as higher temperatures promote lignin depolymerization into smaller fractions that remain soluble during precipitation.

**Figure 1:**
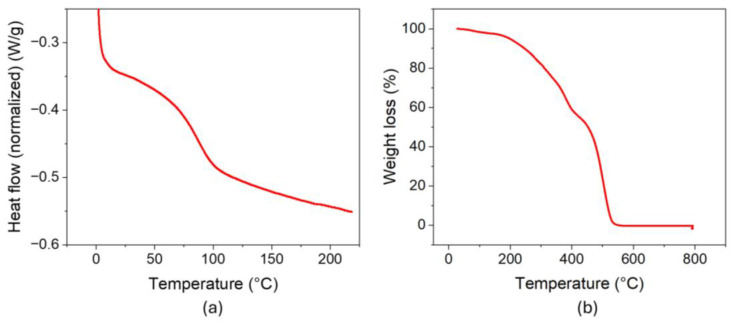
Thermal analysis of the soda lignin obtained: (**a**) DSC thermograms; (**b**) thermogravimetric (TGA) curves.

The thermal behavior was analyzed by differential scanning calorimetry (DSC) and thermogravimetric analysis. DSC thermogram (Figure 1a) revealed a low glass transition temperature (*T*_g_) of 67 °C, which can be attributed to lignin chain degradation at the extraction temperature, leading to smaller fragments that are able to flow at lower temperatures.

The TGA curve of the soda lignin obtained is presented in Figure 1b. An initial mass-loss event occurs in the temperature range of approximately 50–100 °C, primarily associated with the presence of moisture and other volatile components. Subsequently, a second degradation stage was observed between 200 and 300 °C, which is associated with carbohydrates derived from hemicellulose. Finally, a third degradation stage is observed, corresponding to the progressive decomposition of lignin, in which the greatest mass loss takes place. Within the range of approximately 300–450 °C, the process begins with the cleavage of lignin monomer bonds, followed by a gradual mass loss up to 500–550 °C, marking the end of thermal decomposition [17,33]. Beyond this temperature range, no residual mass was observed, indicating the absence of inorganic residues and suggesting that the extracted lignin is predominantly free of inorganic impurities.

**Figure 2:**
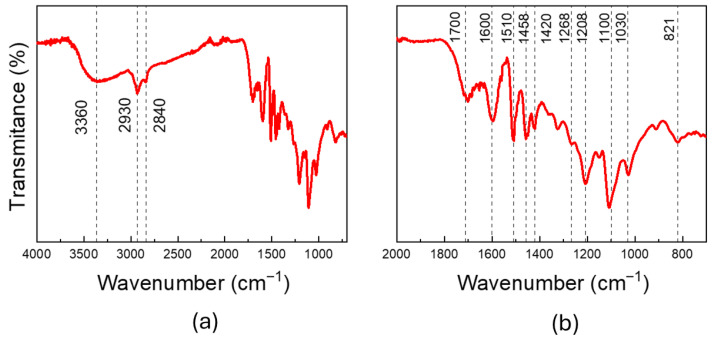
(**a**) FTIR spectra of the obtained lignin; (**b**) magnified view of the 2000–700 cm^−1^ wavenumber region.

The infrared spectrum of the isolated soda lignin is shown in Figure 2a, which exhibits characteristic bands corresponding to functional groups present in lignin. A broad band in the 3400–3200 cm^−1^ region is attributed to O–H stretching of hydroxyl groups, whereas signals at 2930–2920 cm^−1^ arise from C–H stretching in methoxyl substituents. The band centered around 1700 cm^−1^ is ascribed to carbonyl (C=O) stretching, confirming the presence of oxidized functionalities in lignin. Likewise, aromatic rings characteristic of the lignin structure is identified by bands at 1600 and 1510 cm^−1^ [33,34,35,36].

Additionally, an expanded view of the FTIR spectra in the region from 2000 to 700 cm^−1^ is provided in Figure 2b. These spectra highlight several characteristic bands specifically related to lignin monolignols, namely syringyl and guaiacyl units, which were observed in all the samples. These include, among others, the syringyl ring vibration with C–O stretching (around 1321 cm^−1^), the in-plane C–H deformation of the syringyl ring (between 1120 and 1100 cm^−1^), and the C–O stretching vibration of both syringyl and guaiacyl rings (1218–1208 cm^−1^). Furthermore, specific bands from the guaiacyl monolignol were identified at 1268 and 821 cm^−1^ [37,38].

**Figure 3:**
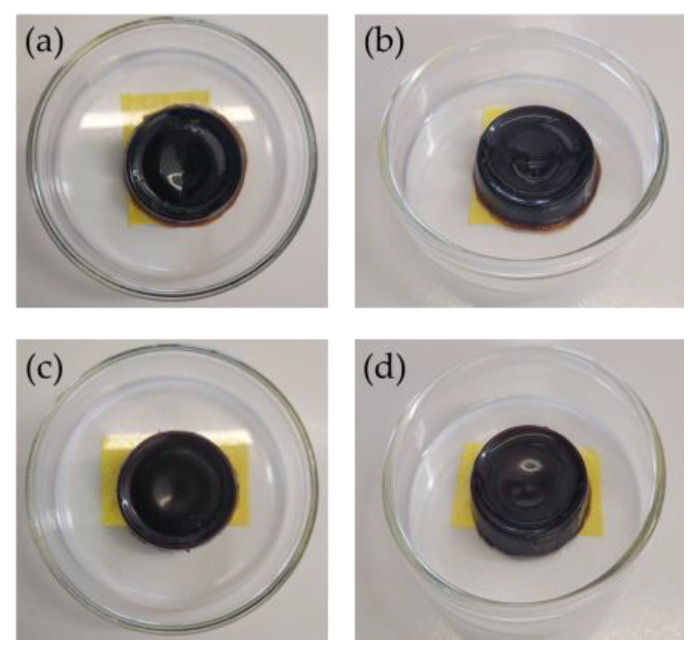
Hydrogels synthesized using the obtained lignin: (**a**,**b**) hydrogel with encapsulated ibuprofen; (**c**,**d**) hydrogel without ibuprofen (blank).

The synthesis of hydrogels from the soda lignin obtained was successfully carried out, incorporating 100 mg of ibuprofen (Figure 3).

**Figure 4:**
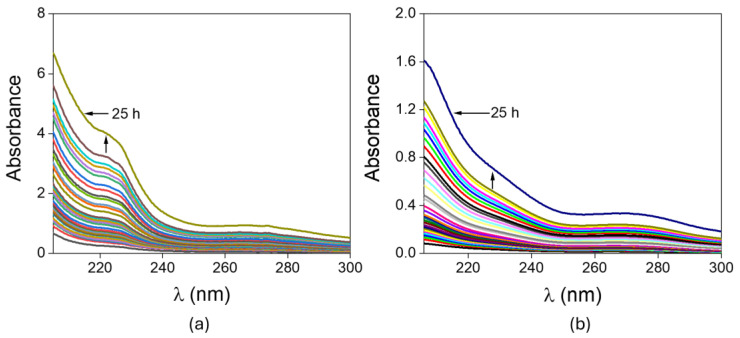
Absorbance measurements made for (**a**) release experiment of the ibuprofen-loaded hydrogel; (**b**) measurements for the blank hydrogel.

Variation in absorbance with time and wavelength for the release experiment conducted using a lignin-based hydrogel tablet loaded with ibuprofen, as well as for the release experiment using an unloaded lignin-based hydrogel (blank), is shown in Figure 4a,b.

**Figure 5:**
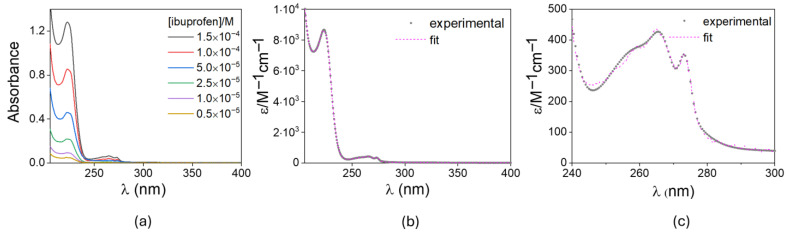
(**a**) Absorbance spectra of several phosphate-buffered (pH 7.0) ibuprofen solutions with concentrations ranging from 10^−6^ to 1.5^−5^ M; (**b**) molar absorptivity was calculated from the spectra and (**c**) zoom of the portion of the spectrum between 240 and 300 nm. The dashed line represents the least-squares fitted molar absorptivity.

Figure 5a shows the spectra of aqueous solutions of ibuprofen recorded at different concentrations of the drug. Figure 5b,c shows the molar absorptivity obtained by fitting the absorbance at each wavelength and its associated concentration to the Beer–Lambert law.

**Figure 6:**
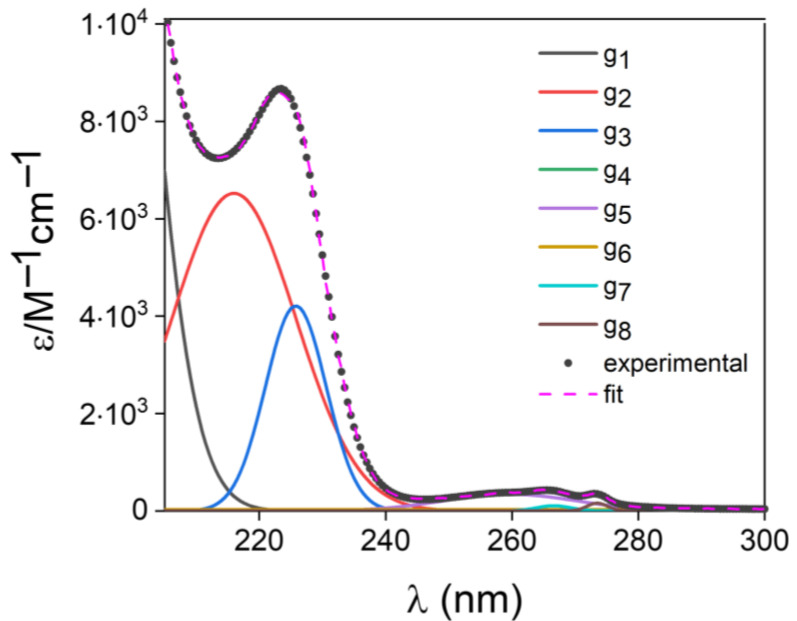
Experimental and least-squares calculated molar absorptivity. The Gaussian function used to model the spectrum is shown.

The average molar absorptivity of ibuprofen was used by the multi-curve response (MCR) procedure to calculate the drug concentration released into the medium (see Supporting Information File). As the MCR algorithm needs an analytical function for the calculation of molar absorptivity, the average spectra in Figure 5b,c was fitted by least squares to the Gaussian series shown in Equation (1),

(1) $$\bar{\epsilon }\left(\lambda \right)=\sum_{i}a_{i}e^{-0.5{\left(\frac{\lambda -\lambda_{0,i}}{s_{i}}\right)}^{2}},$$

where *a_i_* stands for the maximum value of the Gaussian function, *λ*_(0,*i*)_ represents the wavelength at which the maximum is reached, and *s_i_* stands for the Gaussian width at *a_i_*/2. As mentioned above, the Gaussian parameters were estimated by least-squares fitting of *ϵ* (*λ*) to the empirical molar absorptivity values (*ϵ*(*λ*)) by minimizing Equation (2),

(2) $$\bar{\phi }\left(a,\;\lambda_{0},\;s\;\right)=\sum_{k}{\left(\epsilon \left(\lambda_{k}\right)-\bar{\epsilon }\left(\lambda_{k}\right)\right)}^{2},$$

concerning the variable set ($a,\;\lambda_{0},\;s$). The optimized set for the ibuprofen molar absorptivity spectrum is collected in Table 1. Seven Gaussian functions were needed to achieve a correlation coefficient *r* = 0.9999. The Nelder–Mead algorithm, as programmed in the NLOPT library, was used to carry out the minimization process. Figure 6 shows the experimental and calculated molar absorptivity, together with the Gaussian functions.

The drug release profiles were analyzed using the Korsmeyer–Peppas (KP) model coupled with a MCR algorithm to deconvolute the overlapping contributions of ibuprofen and the water-soluble lignin fraction (SLF) from the UV–Vis data, allowing a more accurate interpretation of the ibuprofen release behavior. A detailed description of the MCR procedure, including its schematic representation (Figure S1) and data analysis, is provided in the Supporting Information. Table S1 shows the singular values calculated from the absorbance of experiments 1 and 2, and Figure S2 shows the concentration profile obtained from the release experiments of the unloaded tablet, as derived from the resolved responses and fitted according to Equation (3).

(3) $$c_{i}\left(t\right)=\left(c_{\infty ,i}\times {\;k}_{i}\right)t^{n_{i}},$$

where ${\;k}_{i}$ and $n_{i}$ stand for the KP parameters, $c_{\infty }\;$ is the cumulative concentration at infinite time, and *t* is the release time. Table S2 gathers the fitting results for experiment 1, where the statistical indices indicate that the KP model fits the data adequately. The exponent *n* presented a value of 0.47 for the cylindrical tablet. A value of *n =* 0.45 is indicative of a purely diffusive (Fickian) release mechanism, whereas a value of *n =* 0.89 suggests release by swelling of the support [39]. Thus, the most likely mechanism for the release of the SLF into the media is by diffusion.

**Figure 7:**
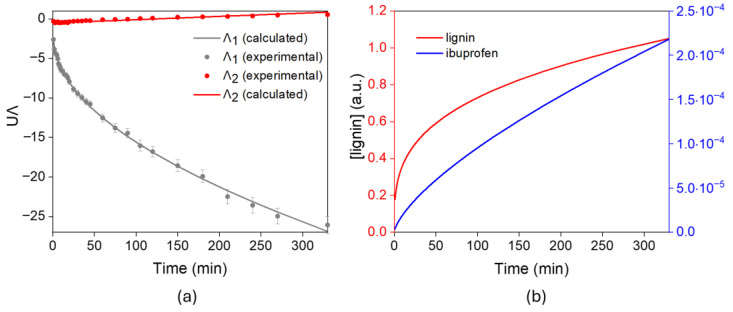
(**a**) Experimental release data of the SLF (red) and ibuprofen (gray) obtained from UV–Vis absorbance measurements (symbols) and KP modelized abstract responses (solid lines) calculated from the absorbance of the ibuprofen release experiment. (**b**) Concentration profiles of SLF and ibuprofen obtained from the MCR-resolved abstract responses (lignin in a.u.).

Figure 7a shows the results from the MCR analysis of the data shown in Figure 5a related to the ibuprofen release (Experiment 2). The figure shows the abstract responses (**A**_u_ = **UA**), whereas Figure 7b shows the concentrations calculated after applying the MCR algorithm using the KP model. As noted above, the concentration curve for the SLF was given in arbitrary units, and that of ibuprofen in molar units. In this case, the molar absorptivity was determined from a standard.

The statistical indices and the values of the constants calculated from the least-squares fit to the KP model are summarized in Table S3. The found correlation coefficient was 0.9995, and only 2% of the variation in absorbance was not explained by the model. Two release curves are presented, one pertaining to SLF and the other to ibuprofen. The results indicate that the mechanism of the lignin release from ibuprofen-loaded tablets differs from that observed in Experiment 1. The value of the exponent *n* = 0.3 of the lignin curves was found to be outside the Fickian (0.45) and the swelling (0.89) limits. The decrease in the SLF release exponent from *n* = 0.47 in the unloaded system to *n* = 0.3 after ibuprofen incorporation suggests a change in the release mechanism of the soluble lignin fraction induced by drug loading. This shift is associated with specific interactions between ibuprofen and lignin, such as hydrogen bonding or hydrophobic interactions with the aromatic domains of soluble lignin fraction, which may restrict lignin mobility within the hydrogel network and result in a more diffusion-limited release behavior. Nevertheless, for ibuprofen, *n* = 0.69 exponent falls inside this interval, suggesting that the delivery process occurs through a combination of mechanisms, with ibuprofen being released during the hydrogel swelling process. The analysis also indicates that the ibuprofen and the SLF are released into the medium at different rates, with the release of the drug being slower.

Additionally, the release profile indicates that 87% of the initial ibuprofen load was released from the hydrogel within 5 h, confirming the successful incorporation of the drug into the hydrogel matrix and highlighting the promising potential of lignin for drug delivery applications. Although direct comparisons with previously reported PVA–lignin hydrogel systems are limited, studies where similar lignin-PVA systems were used for drug delivery, such as lignin–PVA hydrogels, have been used for ciprofloxin delivery, achieving an 88.2% release of the drug after 10 h. Another study with a system lignin–PVA–(Poli(N-isopropylacrylamida)) hydrogels for caffeine encapsulation obtained a 100% release in 5 h. As can be appreciated, the rate obtained is relatively could be considered rapid, depending on the targeted application. This fact could be advantageous for applications where the swift onset of therapeutic action is desirable, such as the management of acute pain or inflammation. Fast-acting ibuprofen formulations have been shown to reach maximal plasma concentrations earlier and provide more rapid pain relief compared to slower formulations, which is clinically relevant for acute conditions [40]. Additionally, initial burst release from hydrogels has been reported as beneficial during the early inflammatory phase of wound healing [41]. However, the release rate could be modulated for controlled medication delivery in future studies by tuning the hydrogel network properties. For instance, the mesh size could be reduced by increasing the cross-linking density through higher cross-linker concentrations or by adjusting the lignin:PVA ratio. Additionally, increasing the PVA content or modifying the lignin structure to introduce a higher density of reactive phenolic groups could promote the formation of additional cross-links, leading to a tighter network and slower drug release [42].

During this comparison between similar systems (PVA–lignin hydrogels), it is important to note that those studies do not apply any modeling approach to resolve the overlapping UV–Vis contributions of the soluble lignin fraction and the drug, which underscores the robustness and added value of the analytical strategy used in this work. In addition, it is noteworthy that throughout the release test, the hydrogel exhibited remarkable resistance properties, as it did not undergo disintegration or rupture. Its ability to absorb water also stands out, highlighting its structural robustness and its capacity to maintain its physical properties during the release process.

## 3. Conclusions

This study demonstrates the valorization of pruning residues through lignin extraction and its application in hydrogel synthesis for drug delivery. FTIR confirmed the presence of characteristic functional groups and monolignols (syringyl and guaiacyl), while TGA indicated a high degree of lignin purity with no detectable inorganic residue. Release profile analysis using the Korsmeyer–Peppas model and multivariate curve resolution showed a good fit for both components and indicated that ibuprofen was released more slowly than the soluble lignin fraction. Ibuprofen exhibited a hybrid diffusion–swelling mechanism, reaching 87% cumulative release. These results highlight the potential of lignin-based hydrogels as advanced matrices for biomedical applications. To advance toward clinical translation, future studies should focus on specific tests based on the targeted biomedical application. In addition, MCR-based deconvolution of drug and lignin release profiles suggests that these systems are well suited for multifunctional co-delivery, pairing anti-inflammatory agents with the inherent antioxidant or antimicrobial properties of lignin, thereby extending their potential applications to regenerative medicine, wound healing and tissue engineering.

## 4. Materials and Methods

Sulfuric acid (95–98%) was obtained from Scharlab (Sentmenat, Barcelona, Spain). Sodium hydroxide (≥98%), poly(vinyl alcohol) (PVA ≥ 99%), ibuprofen sodium salt (≥98%), and epichlorohydrin (≥98%) were purchased from Sigma-Aldrich (Darmstadt, Germany).

### 4.1. Lignin Extraction

Lignin (S/G ratio 0.85, see Figure S3) was extracted from orange trees pruning waste located in the Safor region of Valencia (Spain) using an alkaline extraction method, with the reaction conducted at 190 °C in a 1 M NaOH media with biomass:media ratio 1:10 during 3 h. The lignin fraction was obtained after precipitation with sulfuric acid [43]. Finally, lignin was rinsed several times by centrifugation.

### 4.2. Extraction Percentage

The lignin extraction yield was calculated relative to the initial total dry biomass mass according to the equation below:

(4) $$\%EP=\frac{m_{1}}{m_{s}}\;\times 100$$

In this equation, EP represents the resulting extraction yield, *m*_l_ is the mass of lignin recovered, and *m*_s_ corresponds to the amount of biomass used in the extraction. All parameters refer to dry mass.

### 4.3. Structural Characterization

To identify the functional groups, present in the isolated lignin, Fourier-transform infrared (FTIR) spectroscopy was carried out using an Agilent Cary 630 spectrophotometer (Agilent Technologies, Santa Clara, CA, USA) in transmittance mode over the 4000–500 cm^−1^ range, acquiring 32 scans per sample. The glass transition temperature (*T*_g_) of the extracted lignin was determined by differential scanning calorimetry (DSC) on a TA Instruments (DSC 20, New Castle, DE, USA), where approximately 4 mg of sample was sealed in a standard aluminum pan and heated from 0 to 220 °C at 10 °C/min under a constant nitrogen atmosphere. In addition, thermogravimetric analysis (TGA) was performed using a TA Instruments TGA 550 (New Castle, DE, USA), in which approximately 5 mg of lignin was heated to 900 °C at a rate of 10 °C min^−1^ under an air atmosphere to assess its thermal degradation profile and oxidative stability.

### 4.4. Hydrogel Preparation

**Figure 8:**
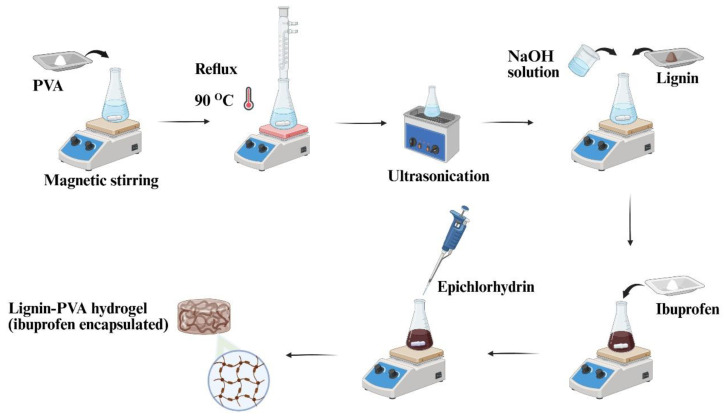
Schematic illustration of the preparation of lignin–PVA hydrogel with ibuprofen encapsulation.

The synthesis of the hydrogel is schematically illustrated in Figure 8 and follows a protocol adapted from a previously reported method [43,44]. Poly(vinyl alcohol) (PVA) was first completely dissolved in water (16 wt%) by heating under reflux at 90 °C for 30 min. After cooling to ambient temperature, 2 mL of the resulting PVA solution, corresponding to 0.32 g of PVA, was used for hydrogel mixture. Subsequently, 1 mL of 2.5 M NaOH and 0.18 g of the isolated soda lignin were added to the PVA solution, and the resulting mixture was stirred for 1 h to obtain a uniform and homogeneous precursor solution. Then, ibuprofen (100 mg, an active pharmaceutical ingredient) was gradually introduced during stirring to enhance its solubility within the polymer matrix. The amount of ibuprofen was selected empirically to evaluate the feasibility of drug loading in the proposed lignin-based hydrogel system. Subsequently, 100 µL of epichlorohydrin (ECH) was added as a cross-linking agent. The solution was then subjected to vigorous stirring for 30 min to facilitate a uniform cross-linking reaction throughout the polymer matrix. The prepared solution was then left undisturbed at ambient temperature for 24 h to allow complete hydrogel formation. Blank hydrogels were prepared using the same protocol, except without the addition of ibuprofen.

### 4.5. In Vitro Drug Release Study

In vitro drug release experiments were evaluated at room temperature in a vessel containing a phosphate-buffered solution (pH = 7) to approximate the physiological pH (7.35–7.45) and to avoid confounding effects arising from pH-induced changes in ibuprofen ionization or hydrogel structure. This approach allows the intrinsic release mechanism of the lignin–PVA hydrogel system to be clearly identified using the KP model supported by MCR analysis. A single hydrogel tablet was submerged and stirred using a submerged magnetic stir bar to ensure agitation while preventing mechanical erosion of the tablet. This setup confirmed that mass transfer was governed solely by dissolution and component diffusion. A preliminary control study (Experiment 1) was performed using a blank hydrogel to account for any spectral interference from the hydrogel matrix. The main study (Experiment 2) utilized the ibuprofen-loaded tablet (100 mg). The maximum theoretical concentrations in the release medium were for ibuprofen and lignin. Aliquots (2 mL) were withdrawn at regular intervals over, with a final reading taken at 25 h.

### 4.6. Drug Quantification and Spectrophotometric Analysis

Aliquots were filtered and diluted prior to analysis. The concentration of released components was determined by measuring the absorbance using a Jasco model V770 double-beam spectrophotometer (Madrid, Spain) equipped with a path length quartz cell. Spectra were acquired over the designated wavelength range at maximum scanning speed. Ibuprofen concentration was quantified using a pre-established calibration curve, which correlates absorbance to concentration. This curve was generated from an aqueous ibuprofen stock solution (10^−3^ M) diluted to standards ranging from 1.5^−4^ M to 1.5^−6^ M. The absorbance of these standards was recorded between 200 and 400 nm to define the calibration relationship, as further detailed in the Results section (Section 2).

## Figures and Tables

**Table 1:** Modeling of ibuprofen molar absorptivity spectrum. Gaussian parameters calculated from minimization of Equation (3).

***i***	$a_{i}$	$\lambda_{0,i}$	$s_{i}$
1	2.129253 × 10^6^	1.934617 × 10^2^	7.721564
2	6.521756 × 10^3^	2.159834 × 10^2^	9.816513
3	4.210365 × 10^3^	2.257779 × 10^2^	4.814559
4	1.583795 × 10^2^	2.905355 × 10^2^	2.165994 × 10^1^
5	3.418569 × 10^2^	2.596958 × 10^2^	1.056078 × 10^1^
6	2.577380 × 10^1^	2.650000 × 10^2^	1.651721 × 10^2^
7	1.072217 × 10^1^	2.665899 × 10^2^	2.747695
8	1.614182 × 10^1^	2.735039 × 10^2^	1.632219

## Data Availability

The data supporting this article are included in the Supporting Information, and the datasets are available from the authors upon reasonable request.

## Supplementary Materials

The following supporting information can be downloaded at https://www.mdpi.com/article/10.3390/gels12020104/s1, Figure S1. Schematic representation of the methodology followed for the KP-MRC modeling of the release test. Figure S2. (a) Experimental (symbols) and KP modeled abstract responses (solid lines) calculated from the absorbance of the SLF release (Figure 5b). (b) Concentration of SLF worked out from the abstract responses (lignin in a.u.). Figure S3. Quantitative ^31^P NMR spectrum of the soda lignin isolated. Table S1. First five singular values of the absorbance matrix; Table S2. Parameters of the KP equation and statistical indices of the least-squares fit performed with the MCR-KP model of the absorbance data from experiment 1; Table S3. Parameters of the KP model equation and statistical indices of the least-squares fit performed with the MCR-KP model of the absorbance data from experiment 2.

## Abbreviations

The following abbreviations are used in this manuscript:

DSC	Differential scanning calorimetry
ECH	Epichlorohydrin
GHG	Greenhouse gases
FTIR	Fourier-transform infrared spectroscopy
KP	Korsmeyer–Peppas
MCR	Multivariate curve resolution
NMR	Nuclear magnetic resonance
PVA	Poly(vinyl alcohol)
SVD	Singular value decomposition
*T*_g_	Glass transition temperature
TGA	Thermogravimetric analysis
UV-VIS	Ultraviolet–visible spectroscopy
