# Supplementary material for: Valorization of Plant Biomass Through the Synthesis of Lignin-Based Hydrogels for Drug Delivery
Source: Gels. 2026 Jan 27;12(2):104. doi: 10.3390/gels12020104 (PMC12939969; doi:10.3390/gels12020104)
Supplement: Supplementary file 1 [file gels-12-00104-s001.zip › gels-4097981-supplementary.pdf]

Supporting information

Valorization of plant biomass through the synthesis of lignin-based hydrogels for drug delivery

Natalia Cárdenas-Vargas[1], Nazish Jabeen[1], Jose Huerta-Recasens[1], Francisco Pérez-Pla[1], Clara M Gómez[1], Maurice Collins[2], Leire Ruiz Rubio[3], Rafael Muñoz-Espí[1] and Mario Culebras[1]*

[1] Institute of Materials Science (ICMUV), University of Valencia, PO Box 22085, E46071 Paterna, 46980, Spain.

[2] Stokes Laboratories, School of Engineering, Bernal Institute, University of Limerick, Limerick, V94 T9PX, Ireland.

[3] Macromolecular Chemistry Group (LQM), Physical Chemistry Department, Faculty of Science and Technology, University of the Basque Country (UPV/EHU), 48940 Leioa, Spain.

*Corresponding author.

E-mail: mario.culebras@uv.es

Results and discussion

Multi-curve response (MCR) analysis of the release experiments

The drug release profiles were analyzed using the Korsmeyer–Peppas (KP) model coupled with an MCR algorithm to deconvolute the overlapping contributions of ibuprofen and the water-soluble lignin fraction (SLF) from the UV–Vis data, allowing a more accurate interpretation of the ibuprofen release behavior. In Figure S1 a schematic representation of the modeling procedure is shown.

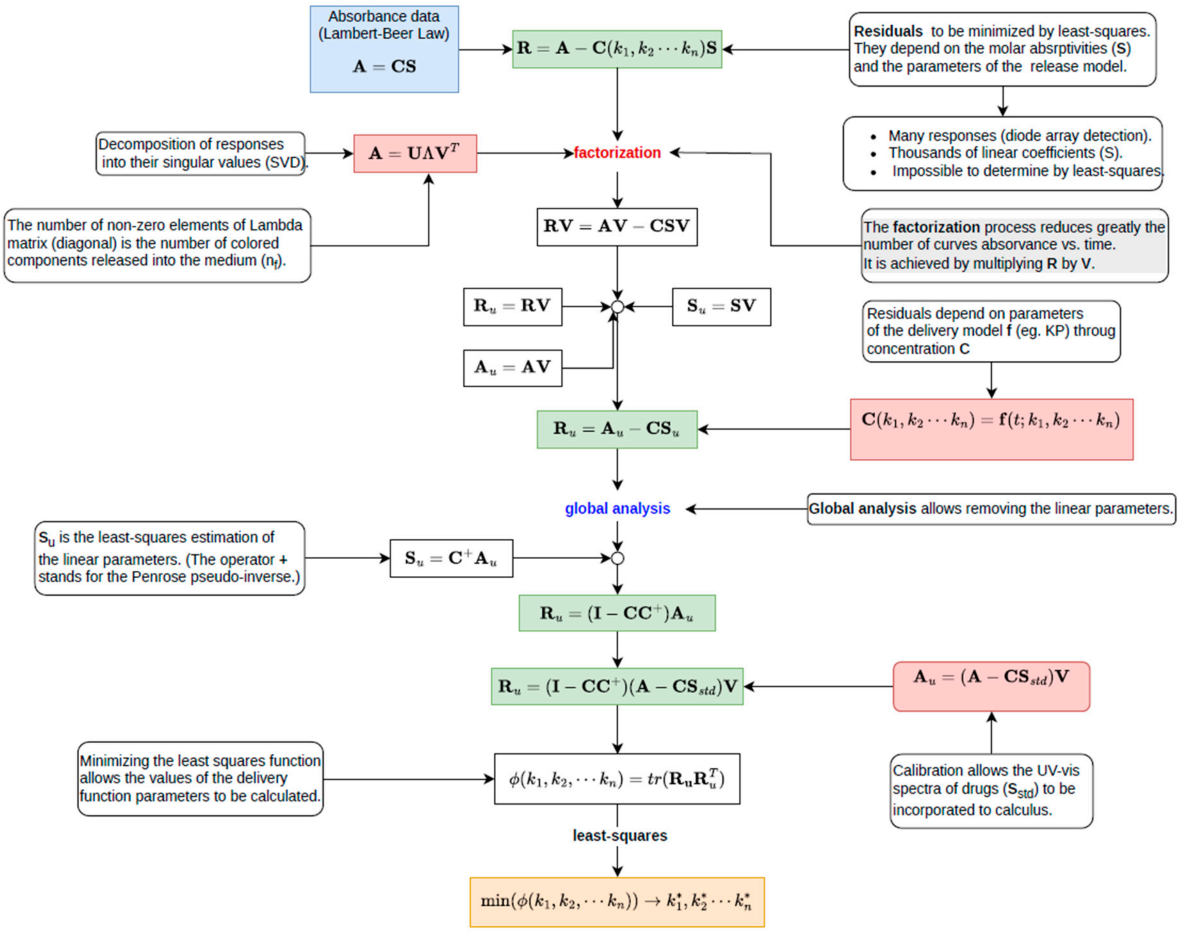

Figure S1. Schematic representation of the methodology followed for the KP-MRC modeling of the release test.

As previously stated in the manuscript, Figure 5a depicts the absorbance recorded from hydrogels loaded with ibuprofen, while Figure 5b illustrates this magnitude measured using unloaded hydrogel tablets. It is noteworthy that the absorbance was recorded within the wavelength range of 205 to 300 nm. The high molar absorptivity of ibuprofen in

wavelength internal under consideration prevented accurate measurement of the absorbance. Therefore, samples were diluted by a factor of four to ensure that the absorbance values fell within the 0-1 range. The figure illustrates the measured absorbance multiplied by four to facilitate comparison of the magnitude for both systems, loaded and unloaded hydrogels.

The singular value decomposition of absorbance indicates that this property can be reconstructed by one or two absorbent species, respectively, for experiments 1 (unloaded hydrogel) and 2 (ibuprofen-loaded hydrogel). Table S1 shows the singular values calculated from the absorbance of experiments 1 and 2. In the case of Experiment 1, a singular value of 17.5 was observed, which was significantly higher than the remaining values, which were approximately equal to the standard deviation of the residuals (7×10^{-2}). Consequently, the four most recent singular values contain minimal kinetic information. It can be concluded that the time variation of absorbance can be reconstructed from the concentration of a single absorbing species, which is likely to be the SLF.

Notwithstanding, two singular values ($\lambda_1 = 72.2$, $\lambda_2 = 2.01$) exceeding the magnitude order to the standard deviation of the residuals (0.23) were obtained for the ibuprofen-loaded hydrogels. Consequently, the time course of the absorbance had to be reconstructed from the time course of at least two absorbent species, namely ibuprofen and the SLF.

Table S1. First five singular values of the absorbance matrix.

Hydrogel	Λ_1	Λ_2	Λ_3	Λ_4	Λ_5	n_f^a
Unloaded	17.5	0.077	0.050	0.020	0.006	1
Ibuprofen	72.2	2.01	0.52	0.16	0.007	2

n_f^a, number of absorbing species.

Figure S2 shows the analysis results obtained when applying the MCR procedure to the data shown in Figure 5b, the release from the unloaded tablet. The figure shows the abstract responses (Au = AV) resulting from the factorization and removal of the array ϵ (i.e., the UV-Vis molar absorptivity spectra) together with the concentration profile calculated from the KP model given by Equation S1,

$$c_i(t) = (c_{\infty,i} \times k_i)t^{n_i} \quad \text{(S1)}$$

In Eq. S1 k_i and n_i stood for the KP parameters, c_∞ is the cumulative concentration at infinite time, and t is the delivery time. Table S2 gathers the fitting results for experiment 1. The concentration curve was presented in arbitrary units, as the calibration of the SLF was not conducted.

Table S2. Parameters of the KP equation and statistical indices of the least-squares fit performed with the MCR-KP model of the absorbance data from experiment 1.

R	LOF	STD error	$c_\infty \times k$	n
0.999_2	2.14	6.9×10^{-2}	4.7×10^4	0.47

The statistical indices indicate that the KP model fits the data adequately. The found correlation coefficient was 0.9995, and only 2% of the variation of absorbance was not explained by the model. Since the value of c_∞ could not be determined, the value of Korsmayer-Peppas constant k could not be evaluated either. The exponent n presented a value of 0.47 for the cylindrical tablet, a value of $n = 0.45$ is indicative of a purely diffusive (of Fickian) release mechanism, whereas a value of $n = 0.89$ suggests release by swelling of the support. Thus, the most likely mechanism for the release of the SLF into the media is by diffusion.

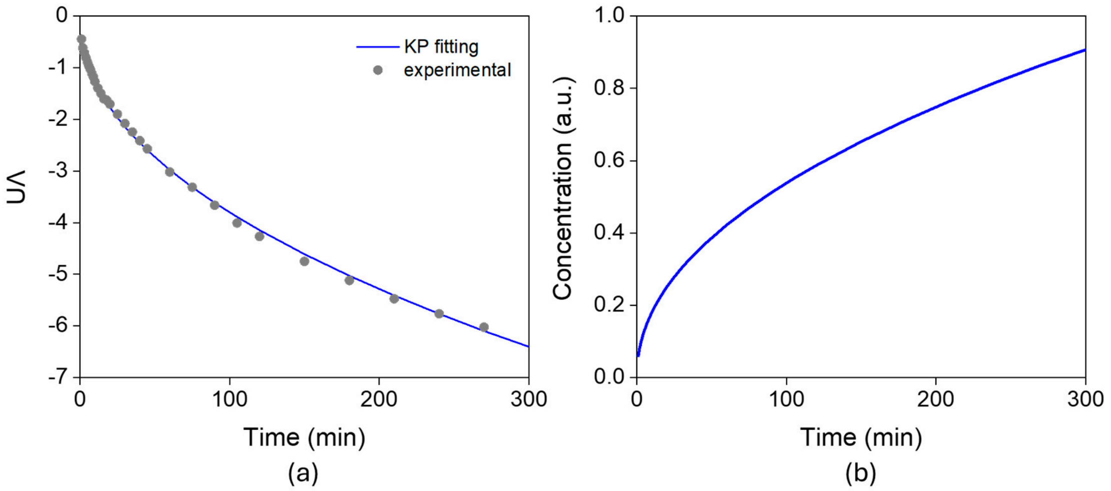

Figure S2. (a) Experimental (symbols) and KP modelized abstract responses (solid lines) calculated from the absorbance of the SLF release (Figure 5b). (b) Concentration of SLF worked out from the abstract responses (lignin in a.u.).

Table S3. Parameters of the KP model equation and statistical indices of the least-squares fit performed with the MCR-KP model of the absorbance data from experiment 2.

	R	LOF	STD error	$c_{\infty} \times k$	n
	0.999_5	2.48	2.35×10^{-1}		
Ibuprofen				3.96×10^{-6}	0.69
Lignin				7.74×10^{4}	0.3

Data Analysis

Statement of the problem

The basis of data analysis in the Lambert-Beer law (Eq. S2),

$$\mathbf{A}(\mathrm{n_t}, \mathrm{n_r}) = \mathbf{C}(\mathrm{n_t}, \mathrm{n_s})\mathbf{S}(\mathrm{n_s}, \mathrm{n_r}) \tag{S2}$$

Where **A** is the array of absorbances, and **C** and **S** are arrays storing the molar concentration and the molar absorptivity coefficients of the release species, in this case lignin and ibuprofen. The elements of **C** are computed by using one of the delivery functions that are described in the literature,[1–3] such as the KP-model shown in Eq. (S1). Dimensions n_t, n_r, and n_s stand for the number of measurements (i.e. number of times at which the absorbance has been measured), the number of observation channels (i.e. the number of wavelengths at which absorbance has been measured), and the number of UV-vis absorbing species respectively.

The purpose of the hard-modeling analysis is the determination of the delivery coefficients associated with the model, in this case, the KP parameters *k* and *n*, by means of the least-squares method. Regression analysis consists of minimizing the values of the residuals (**R**) regarding the above mentioned variables. The residuals are defined by Eq. S3,

$$\mathbf{R}(\mathbf{k},\mathbf{n},\mathbf{S}) = \mathbf{A} - \mathbf{C}(\mathbf{k},\mathbf{n})\mathbf{S} \quad \text{(S3)}$$

where $\mathbf{A}$ is the experimental absorbance array, and the product $\mathbf{CS}$ is the calculated absorbance estimated from the delivery model. Note that residuals are a nonlinear function of the delivery model parameters and linearly dependent on molar absorptivity coefficients.

The number of variables for which the residuals must be minimized can be overwhelming. For example, if we realize the system consists of 2 absorbing species (e.g. lignin and ibuprofen), and the absorbance is measured at 100 wavelengths, the residuals will depend on 200 molar absorptivity coefficients, and 2 values of k and n, making 204 variables in total. Obviously, the number of molar absorptivity coefficients is too large to perform the minimization successfully. However, the goal of the calculation only depends on 4 variables, namely k_1, k_2, n_1, and n_2.

To solve this issue, the array **A** is factored[29] using the Singular Value Decomposition (SVD) technique,[4] which allows for reduction of the n_r value. After factorization, array **S** is removed from Eq. S3. The algorithms that perform both tasks are called Multivariate Curve Resolution (MCR) methods. It is important to highlight the term "resolution", since because of array **A** analysis, it is possible to work out the change with time of concentration of species involved in the delivery process, together with their molar absorptivity spectra. Below, the two steps of the procedure, factorization and ***S*** array removal, are briefly explained.

As indicated above, the factorization is carried out using the SVD algorithm. In principle, any two-dimensional array can be written as the matrix product shown in Eq. S4, where (^{T}) denotes the transpose matrix operator,

$$\mathbf{A}(n_t, n_r) = \mathbf{U}(n_t, n_r)\mathbf{\Lambda}(n_r, n_r)\mathbf{V}^T(n_r, n_r) \quad \text{(S4)}$$

The **U** and **V** arrays have the property of being orthonormal (i.e., $\mathbf{UU}^T = \mathbf{1}$, $\mathbf{VV}^T = \mathbf{1}$), and they are known as the *abstract concentration* and *abstract spectra* arrays, respectively. The diagonal **Λ** array contains the singular values of **A**.

The analysis of array $\boldsymbol{\Lambda}$ reveals that the first n_f elements of the main diagonal have very high values, the rest of them being very small. In fact, they are not null due to the experimental uncertainty of absorbance measurements. This means that Eq. S4 can be rewritten as,

$$\mathbf{A}(n_t, n_f) = \mathbf{U}(n_t, n_f)\boldsymbol{\Lambda}(n_f, n_f)\mathbf{V}^T(n_r, n_r) \quad \text{(S5)}$$

From a mathematical point of view, the value of n_f is equal to the rank of array $\mathbf{A}$, that is, it is equal to the smallest number of linearly independent vectors that allow to reconstruct this matrix. The value of n_f is always less than or equal to n_s, and therefore much less than n_r (i.e. $n_f \le n_s << n_r$). For the system under study, the value of $n_f = n_s$ coincides with the number of UV-vis absorbing species.[5]

We now apply the SVD technique to reduce the size of array $\mathbf{A}$, and thus the size of matrix $\mathbf{R}$. It suffices to multiply by the right Eq. S3 by $\mathbf{V}$,

$$\mathbf{R}_{\boldsymbol{u}} = \mathbf{RV} = \mathbf{AV} - \mathbf{C}(\mathbf{SV}) = \mathbf{A}_{\boldsymbol{u}} - \mathbf{CS}_u \quad \text{(S6)}$$

Note that the dimensions of $\mathbf{R}$ were (n_t, n_r), while those of $\mathbf{R}_u$ are (n_t, n_f). The size reduction achieved has been substantial. The second step consists of eliminating the linear parameters.[6] This is achieved by replacing the array $\mathbf{S}_u$ in Eq. S6 by its least squares estimate, $\mathbf{S}_u = \mathbf{C}^+\mathbf{A}_u$,

$$\mathbf{R}_{\mathbf{u}}(\mathbf{k}, \mathbf{n}) = (\mathbf{I} - \mathbf{CC}^+)\mathbf{A}_{\mathrm{u}} \quad \text{(S7)}$$

where $\mathbf{C}^+ = (\mathbf{C}^T\mathbf{C})^{-1}\,\mathbf{C}^T$ stands for the Penrose pseudo-inverse array (operator ($^+$)), and I is the identity matrix. This algebraic manipulation makes the residuals to depend only on the parameters related to the delivery model. Finally, the kinetic parameters are calculated by minimizing the objective least-squares function, where (tr) is the trace matrix operator,

$$\phi(k, K) = tr(\mathbf{R}_u, \mathbf{R}_u^T) \quad \text{(S8)}$$

However, the concentration curves obtained are relative if the molar absorption spectra are not included in the calculations. Let us see how the above equations should be modified to include the spectra of standards. First, it will be assumed that not all species are calibrated. In this case, the absorbance is expressed as the sum of contributions done by uncalibrated and calibrated species,

$$\mathbf{A} = \mathbf{CS} + \mathbf{C}_{\mathrm{s}}\mathbf{S}_{\mathrm{s}} = \mathbf{CS} + \mathbf{A}_{\mathrm{s}} \tag{S9}$$

where $\mathbf{S}_{\mathrm{s}}$ designates the standard spectra, and $\mathbf{C}_{\mathrm{s}}$ and $\mathbf{C}$ the concentration arrays associated with calibrated and uncalibrated species. The part of the response associated with the uncalibrated absorbing compounds is given by,

$$\mathbf{A} - \mathbf{A}_{\mathrm{s}} = \mathbf{CS} \tag{S10}$$

In order to achieve the linear independence of responses, Eq. (S10) is multiplied by **V**, which results in expression Eq. (S11),

$$\mathbf{AV} - \mathbf{A}_{\mathrm{s}}\mathbf{V} = \mathbf{CSV} \tag{S11}$$

Eq. (S11) is rewritten as,

$$\mathbf{A}_{\mathrm{u}} - \mathbf{A}_{\mathrm{u,s}} = \mathbf{CS}_{\mathrm{u}} \tag{S12}$$

Where $\mathbf{A}_{\mathrm{u,s}} = \mathbf{A}_{\mathrm{s}}\mathbf{V}$ and $\mathbf{S}_{\mathrm{u}} = \mathbf{SV}$. Therefore, the modified expression for residuals is given by Eq.(S13),

$$\mathbf{R}_{\mathrm{u}} = \mathbf{A}_{\mathrm{u}} - \mathbf{A}_{\mathrm{u,s}} - \mathbf{CS}_{\mathrm{u}} \tag{S13}$$

Substitution of the least-squares estimation of the abstract unknown spectra ($\mathbf{S}_{\mathrm{u}} = \mathbf{C}^{+}(\mathbf{A}_{\mathrm{u}} - \mathbf{A}_{\mathrm{u,s}})$) in Eq.(S13) results in the expression for residuals currently wanted,

$$\mathbf{R}_{\mathrm{u}} = (\mathbf{I} - \mathbf{CC}^{+})(\mathbf{A}_{\mathrm{u}} - \mathbf{A}_{\mathrm{u,s}}) \tag{S14}$$

If all species were calibrated, Eq.(S9) reduces to Eq.(S15)

$$\mathbf{A} = \mathbf{CS}_{s} \tag{S15}$$

And therefore, the expression for the residuals, which considers the linear independence of abstract responses, is given in Eq.(S16)

$$\mathbf{R_u} = (\mathbf{A} - \mathbf{CS_s})\mathbf{V} \tag{S16}$$

The calculations were carried out using the DELIVERYMCR algorithm programmed in the Julia language (unpublished).[7] The program was written by Professor F. Perez-Pla, member of the ICMUV staff.

Determination of the syringyl/guaiacyl (S/G) ratio

Quantitative ^{31}P NMR experiments were conducted on the isolated lignin using a Bruker Neo 500 MHz spectrometer, according to the protocol described by Meng et al.[8]

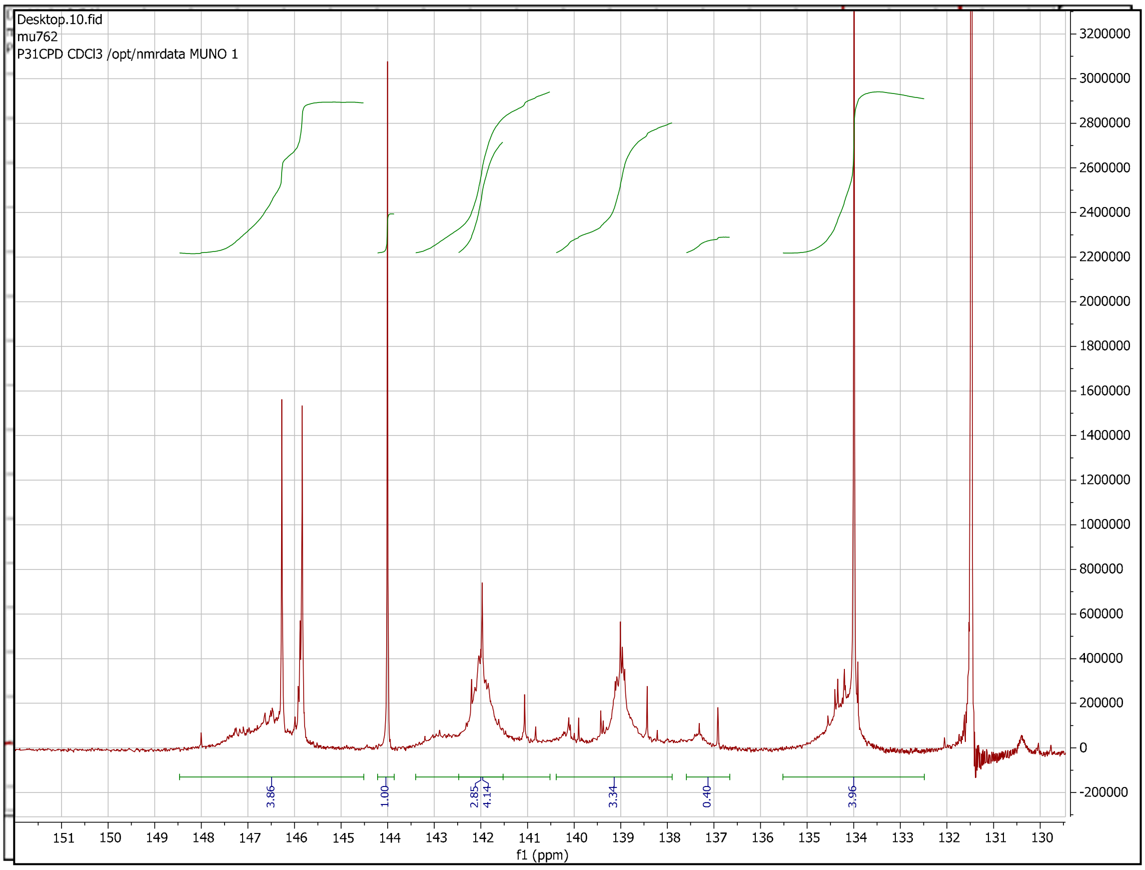

Figure S3. Quantitative ^{31}P NMR spectrum of the soda lignin isolated.

References

(1) Grassi, M.; Grassi, G. *Mathematical Modelling and Controlled Drug Delivery: Matrix Systems*. Current Drug Delivery. http://www.ncbi.nlm.nih.gov/pubmed/16305412.

(2) Dash, S.; Murthy, P. N.; Nath, L.; Chowdhury, P. Kinetic Modeling on Drug Release from Controlleddrug Delivery Systems. *Acta Pol Pharm.* 2010, *67*, 217–223.

(3) Siepmann, J.; Siepmann, F. Mathematical Modeling of Drug Delivery. *J. Pharmaceut.* December 8, 2008, pp 328–343. https://doi.org/10.1016/j.ijpharm.2008.09.004.

(4) Press, W. H.; Teukolsky, S. A.; Vetterling, W. T.; Flannery, B. P. Numerical Recipes in C : The Art of Scientific Computing; Cambridge University Press, 1992; pp 456–495.

(5) Meloun, M.; Syrový, T.; Vrána, A. Determination of the Number of Light-Absorbing Species in the Protonation Equilibra of Selected Drugs. *Anal Chim Acta* 2003, *489* (2), 137–151. https://doi.org/10.1016/S0003-2670(03)00761-X.

(6) Bonneau, R.; Wirz, J.; Zuberbuhler, A. D. Methods for the Analysis of Transient Absorbance Data (Technical Report). *Pure Appl. Chem* 1997, *69*, 979–992.

(7) Bezanson, J.; Karpinski, S.; Shah, V. B.; Edelman, A. Julia: A Fast Dynamic Language for Technical Computing. 2012.

(8) Meng, X., Crestini, C., Ben, H. *et al.* Determination of hydroxyl groups in biorefinery resources via quantitative ^{31}P NMR spectroscopy. *Nat Protoc* **14**, 2627–2647 (2019). https://doi.org/10.1038/s41596-019-0191-1.
